# Impact of health education intervention on demand of women for cervical cancer screening: a cluster-randomized controlled trial

**DOI:** 10.1186/s13063-022-06765-0

**Published:** 2022-10-01

**Authors:** Gerezgiher Buruh Abera, Solomon Mekonen Abebe, Abebaw Gebeyehu Worku

**Affiliations:** 1 School of Nursing, College of Health Sciences, Mekelle University, Mekelle, Tigray Ethiopia; 2 Institute of Public Health, College of Medicine and Health Sciences, University of Gondar, Gondar, Ethiopia; 3 Amhara Regional Health Bureau, Bahir Dar, Amhara Ethiopia

**Keywords:** Demand, Cervical, Cancer, Screening, Cluster, Randomization

## Abstract

**Background:**

Cervical cancer is considered preventable disease, though it is the second largest killer of women’s cancer in low and middle-income countries. Despite the government’s attempts to broaden screening facilities, the screening service utilization was poor. Our study evaluated the impact of health education intervention on women’s demand for cervical cancer screening.

**Methods:**

Community-based cluster-randomized controlled trial was conducted in thirty district towns as clusters in Tigray region, Ethiopia. A total of 700 women aged 20 to 60 years were recruited for both groups using simple random sampling from April to July, 2018. After baseline data collection, health education intervention was given to the intervention group by trained health professionals using power point presentation and peer group discussion at the nearest health institution. The health education was given for three days followed by subsequent consultations for 6 months. The outcome variable was demand of women for cervical cancer screening. The intent-to-treat and per-protocol analysis were considered to evaluate the inflation of the loss to follow-up on effect size. Chi-square test was used to assess the difference of variables between control and intervention groups at baseline data. Finally, difference in difference analysis was used to see the true effect of the intervention on outcome variable.

**Results:**

A total of 674 participants (340 in intervention and 334 in control groups) were able to complete the follow-up, making a response rate of 96.3%. At baseline, the differences in proportion of all outcome variables in control and intervention groups were not statistically significant. After follow-up, a statistically significant difference between control and intervention groups was observed in the proportion of willingness to screen (*p* value = 0.000), having plan to screen (*p* value = 0.000), ever screened (*p* value = 0.000), and the overall demand for cervical cancer screening (*p* value = 0.000). Finally, the impact of intervention was explained by the difference in differences in the proportion of willingness to screen (36.6%) (*p* value < 0.000), having plan to screen (14.6%) (*p* value < 0.000), ever screened (16.9%) (*p* value < 0.000), and overall demand for cervical cancer screening (36.9%) (*p* value < 0.000).

**Conclusion:**

This study revealed that health education intervention could increase in overall demand of women for cervical cancer screening. Thus, it would be helpful to consider health education in health planning and service provision.

**Trial registration:**

The registration number is PACTR201808126223676; date registered: 23 April 2018, and the type is “retrospectively registered.”

**Supplementary Information:**

The online version contains supplementary material available at 10.1186/s13063-022-06765-0.

## Background

Cervical cancer is a malignant reproductive organ tumor, which manifests itself in the transformation zone of the exo-cervical opening [1]. Demand for screening is an expressed or felt needs of women for cervical cancer screening [2]. Though cervical cancer is the second most devastating cancer in low- and middle-income countries in the world, it is considered preventable disease [1, 3]. Health education interventions promote not only perception of women about cervical cancer but also increases demand for cervical cancer screening [4].

Different studies used a variety of culturally tailored health promotion interventional modalities to increase cervical cancer screening. A study in Barcelona, 2017, showed an increase in demand of cervical cancer screening by 20% in the intervention group, with a difference in differences of 11% [5]. Similarly, a study in southeast Nigeria, 2017, showed an increase in cervical cancer screening practice by 6.8% from pre to post intervention [6].

A health education intervention based studies done in Nigeria, Cameroon, and Enugu state showed that there was an increase in proportion of having good perception (from 5.1 to 95.1%), screening practices (from 4.3 to 8.3%), and very good knowledge (from 2 to 70.5%), and intention to screen (from 70.1 to 75.5%) [7–11]. Other studies in America and Canada showed that there were an increase in screening rate (from 41.1 to 53.4%) in the intervention group, more than the usual-care arm (increased from 25.2 to 34%), with a difference in differences (DID) of 8.8% and an increase in screening rate (from 65.8 to 81.8%) in the intervention group greater than the control group (increased from 70.1 to 75.5%), with a difference in differences of 10.6% respectively [10, 12].

Related to other intervention methods, there was a significant difference in intention of screening between the intervention and control groups using an invitation letter for screening as intervention (with a difference of 9.2%) and phone reminders as intervention (with a difference of 31.4%) [13]. A cervical health literacy study showed that the intervention group experienced significant changes on 7 out of 8 domains, which was more than the control group, that was showed significant change on 1 out of 8 domains of the cervical health literacy [14].

Cervical cancer screening campaign as an intervention method was showed an increase in proportion of screening (from 73 to 87%) in the intervention group, greater than the control group (from 67 to 60%) [15]. About 72% and 39% of participants were also reported having plan to screen and being screened for cervical cancer in the intervention group, greater than the control group (48% and 15%), respectively [16]. Moreover, following intervention, an increase in cervical screening practice was observed from 73% in the pre intervention to 87% in the post intervention [17].

Nevertheless, other studies showed no significant differences in demand for cervical screening between the intervention and control groups [8, 18]. Similarly, the difference in a mean score of screening intent was not statistically significant [13]. Using modified invitation letters as intervention also did not increase the attendance of cervical cancer screening [with a difference of 1.3%; 95% CI (0.3, 2.9)] [19].

Many predictors were associated with demand of women for cervical cancer screening. As shown from studies in Nigeria, Latin America, and Caribbean Countries, variables like marital status, level of education, employment status, ethnicity, fear or embarrassment, and parity were associated with cervical cancer screening intention [6, 20]. Other variables associated with the demand of cervical cancer screening were sources of information, awareness of the test, preventability of the disease, and living in rural areas [21–23].

Though the Ethiopian government health policy focuses on expansion of cervical cancer screening using visual inspection with acetic acid (VIA) [24], only 2% of health institutions were providing cervical cancer screening service, and the service utilization by eligible women remains as low as 0.8% in 2018 [25]. A community-based demand creation program or health education intervention study was not done so far in the country at large and in Tigray regional state in particular.

We hypothesize that a health education intervention could improve the demand of adult women for cervical cancer screening. Therefore, the aim of this study was to decipher the impact of health education intervention on demand of women for cervical cancer screening.

## Methods

The format of this report is prepared based on the CONSORT 2010 guideline for title, abstract, methods, results, discussion and conclusion, which was updated in 2018 for cluster randomized trial (annexed with manuscript submission) [26].

### Study design

A two-arm cluster-randomized controlled trial (CRCT) was conducted in Tigray regional state of Northern Ethiopia. District towns treated as clusters were randomized to intervention and control groups on a 1 to 1 ratio, and enrollment was done from April to July, 2018.

### Participants

This study was conducted in Tigray region, which is located in Northern Ethiopia. Based on the federal population projection of 2014–2017, there was a population of 4,960,003 in the region and a total of 52 districts (34 rural and 18 urban) [27]. Thirty (15 each) out of 52 district towns were selected based on the merits of providing cervical cancer screening services and randomized to either intervention or control group. Out of 1000 participants registered at baseline in both groups, a total of 700 (350 each) eligible participants with the age range of 20 to 60 years, who were sexually active and had no hysterectomy were identified. Those who were diagnosed with cervical cancer at the time of recruitment were excluded. Potential participants were approached for data collection at their home (place of residence).

### Patient and public involvement

No patient or public identifiers were involved in this study.

### The intervention

A health education was given to participants in the intervention group at the nearest health institution of the selected district towns after baseline data collection. The intervention was delivered in a culturally-tailored environment with local language-based (Amharigna and Tigrigna) power point presentations, pictures, and fact sheets. The contents of health education intervention include knowledge of cervical cancer, risk factors, sign and symptoms, service accessibility, method of prevention, side effects, follow-up, and the prognosis. The educational tool was taken from the Federal Democratic Republic of Ethiopia, Ministry of Health Cervical Cancer Prevention and Control Guideline, 2015 [1]. The health education intervention was given for a total of 3 days presentation and peer group discussion and 6 months client’s need-based consultancy follow-up by trained health extension workers in each site.

The education was arranged in such a way that the session of susceptibility was followed by risk factors and the seriousness of the problem and then prevention methods. Finally, screening opportunity, barriers to screening, and possible solutions were discussed.

**Fig. 1:**
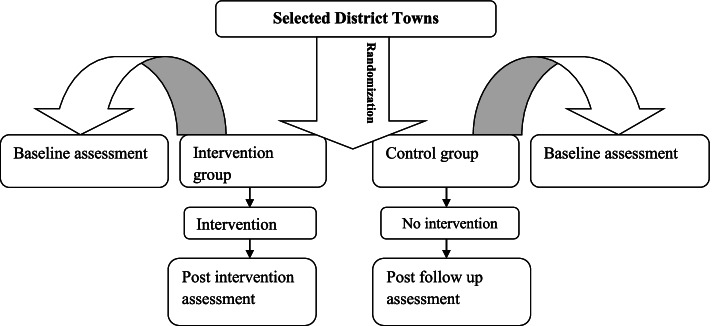
The intervention implementation flow diagram

In the control arm, no health education intervention was given. The “treatment as usual” (TAU) was a comparator condition in the control group, which is the routine health promotion and support of professionals or media given to all residents (Fig. 1).

### Outcomes

The outcome variable was “demand of women for cervical cancer screening.” Demand was defined as a felt or an expressed need, often the individual’s perceptions of variations from normal health and/or expressed in action [28]. It was measured at the individual level before and after intervention. Those who at least say “yes” for either of the willingness, having plan to screen within the next 6 months, and ever screened items were considered as having demand for cervical cancer screening. The overall demand is the union of these items. For those who reported screened, the document was checked from nearby institutions or appointment cards.

### Sample size

The sample size was determined using the two-population proportion formula with the assumption of 0.05 α and 80% power. The proportion of demand was 53.4% in the intervention group and 34% in the control group [28]. A confidence level of 95% and one to one ratio of control to intervention group was used. Since the response from within group participants is unlikely to be independent, a 0.05 intra-cluster correlation coefficient (ICC) was used to increase the sample size [17, 29]. Assuming unequal size clusters, we used the minimum and maximum cluster size to evaluate the coefficient of variation and feasible number of clusters per arm which was checked using the formula *k* > nIρ [30], where *k* is a one arm number of clusters.

Using STATA 14, power and sample size calculator, the sample size calculated was 204. Multiplying with the inflation factor of 2.95 and considering none responses rate of 15%, the final sample size was estimated to be 700 in both groups.

### Randomization

**Fig. 2:**
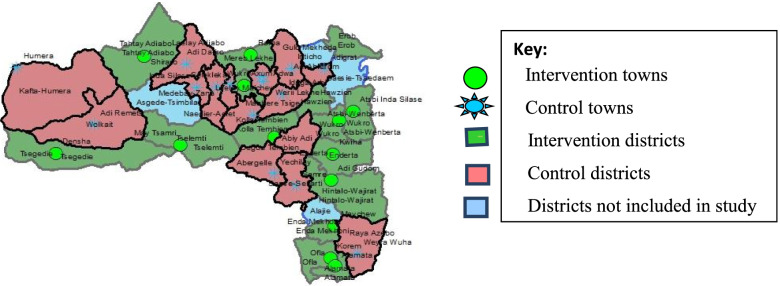
The cluster distribution in the map of Tigray region, Ethiopia. Note: In the map, some districts did not appear, because some districts are newly established and the online map of Tigray region is not updated

Thirty clusters were randomized into either group to minimize contamination between individuals. The clusters were district towns which have villages between them as natural buffering zones (Fig. 2). The selected clusters were assessed for structure of health institutions. Two of the towns have comprehensive specialized hospitals, ten have general hospitals, and 18 have primary hospitals. Based on the level of health facilities, the clusters were stratified in three blocks. The strata were assigned to intervention and control groups using a predetermined one to one order block randomization.

The randomization was done by a computer-based random number generation for each stratum using the list of the clusters from South to Northwest of the region chosen randomly. The random allocation process was done by a person who is not a member of this research. Each district authority provided permission to include clusters in the study. Based on the eligibility criteria, a lists of participants were used from the health extension workers at baseline and simple random sampling was used to recruit study participants from each clusters. Participants were consented to participate in the study. Training providers, follow-up consultants, data collectors and participants did not know whether they were in the intervention group or control group. Furthermore, the waitlist control approach neutralizes the perception of trainers and data collectors that participants may be in either group.

### Statistical methods

The per-protocol (PP) and intention-to-treat (ITT) analyses approaches were used to see the influence of loss to follow-up on effect size in both groups. Chi-square test with *p*-value of < 0.05 was used to evaluate the differences of independent variables in control and intervention groups at baseline and end line. Finally, difference in differences analysis was used to see the true effect of the intervention on the outcome variables.

### Model specification

Based on the impact evaluation with panel data [31], two features are required in which intervention and control groups are compared: (1) there should be intervention and control groups, and (2) at least one observation before and one observation after intervention should be conducted. Therefore, the impact of the intervention was estimated by subtracting the difference in proportion between control and intervention groups at baseline from the difference in proportion between the groups at end line, which is called differences in difference (DID).

We used the following notations: *D*^c^_0_ as demand of the control group in the baseline, *D*^t^_0_ as demand for intervention group at baseline, *D*^c^_1_ as demand of the control group in the end line, and *D*^t^_1_ as demand for intervention group at the end line.

1 $$\mathrm{DID}=\left({D}^t1-{D}^c1\right)-\left({D}^t0-{D}^c0\right)$$

The equation of this model regresses the outcome variable in relation to the intervention, period, and an interaction (intervention*period) variables, as well as fitted with the linear probability model (LPM) equation:

2 $${Demand}_i={\upbeta}_0+{\upbeta}_1\mathrm{Treated}+{\upbeta}_2\mathrm{Period}+{\upbeta}_3\mathrm{Treated}\ast \mathrm{Period}+{\upvarepsilon}_{\mathrm{it}}$$

Based on Eqs. (1) and (2):

3 $${\displaystyle \begin{array}{l}\mathrm{DID}=\left[\left({\upbeta}_0+{\upbeta}_1+{\upbeta}_2+{\upbeta}_3\right)-\left({\upbeta}_0+{\upbeta}_2\right)\right]-\left[\left({\upbeta}_0+{\upbeta}_1\right)-\left({\upbeta}_0\right)\right]\ \\ {}\mathrm{DID}={\upbeta}_3\end{array}}$$

Following the linear probability model, “collapse command” was used to estimate the 2 by 2 table that explain the proportion of demand in both groups over time to develop the difference in differences graph.

### Variables

*Dependent variable*: demand of women for cervical cancer screening

*Independent variables:* (a) socio-demography of the women (age, educational level, marital status, occupation, ethnicity, and religion), (b) reproductive related data (age at first sex, number of partners, gravidity, contraceptive use and abortion), and (c) lifestyle and health (alcohol use, smoking, chronic drug use, and STI history)

*Intervention* is a dummy variable denoted by “0” indicating no health education intervention and “1” indicating that health education intervention is given

*Time* is a dummy variable denoted by “0” indicating the time before intervention and “1” indicating the time after the intervention

*Impact indicator:* The interaction variable calculated from the intervention and time variables, donated as the coefficient of the difference in difference

*Knowledge* is awareness of cervical cancer related conditions, including risk factors, human papilloma virus (HPV) transmission, sign and symptoms, and prevention methods. It was measured using a total of 19 items with one-point score each for “yes” responses, and those scored > 50% were categorized as knowledgeable; otherwise, they were categorized as less knowledgeable.

*Attitude* is the perception of participants with six items—related to attitude on cervical cancer. It was measured based on the six-point Likert scale scored from “0” (strongly disagree) to “5” (strongly agree). Those having a mean score of > 60% were considered as having a supportive attitude; otherwise, they were categorized as less supportive attitude.

### Data collection method

Data were collected using an interviewer administered questionnaire to get baseline information. Follow-up data collection was conducted using the same questionnaire in both groups 6 months after randomization to ascertain changes in participants’ demand for cervical cancer screening. The questionnaire was prepared in English and translated into the local language of Tigrigna and translated back to English by professionals in order to keep consistency of the data.

Training was provided to the selected data collectors for two days about the objective and the process of data collection. Pre-testing was done on 5% of the questionnaires in similar communities to play the role as the study population and this was done in an area that was not included in this study. Close supervision was undertaken during data collection by checking 10% of the filled questionnaires for completeness and accuracy at the closing of each day.

## Results

**Fig. 3:**
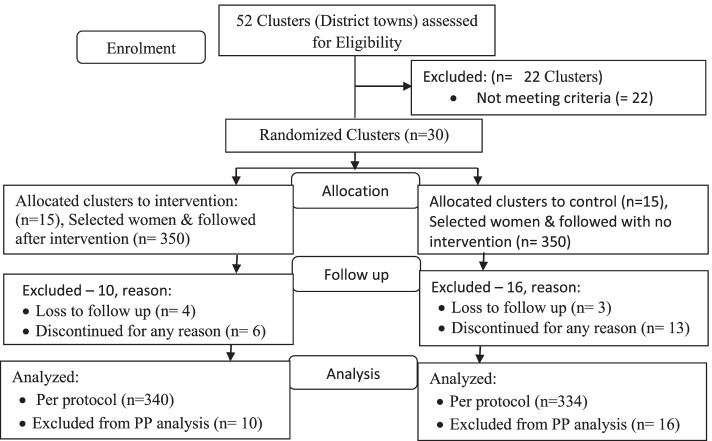
Overview of the schematic representation and health education intervention follow-up (format taken from CONSORT 2010 flow diagram)

A total of 700 eligible participants (350 in control and 350 in intervention groups) were recruited for follow-up, of which 674 (340 intervention and 334 control) were able to complete the follow-up data collection with a response rate of 96.3%. Lost to follow-up rates during 6 months was 4.6% in the control and 2.9% in the intervention group. The reason given to “why they did not respond” was withdrawal or denied [14], health problems [5], and lost with no reason [7] (Fig. 3).

### Recruitment

The study enrollment occurred between April and July 2018 in both groups. Both intervention and control groups were followed for 6 months, which was assumed that it is sufficient time to bring change in health seeking behavior.

### Socio-demographic characteristics of participants

All of the socio-demographic variables showed no significant differences between control and intervention groups at baseline and end line data, except marital status, which showed significant differences both at baseline and end line data.

The mean (± SD) age of the participants at baseline was 32.48 (± 8.9) in the control and 32.97 (±8.5) in the intervention group, and at end line, it was 32.42 (± 9.03) in the control and 33.04 (± 8.44) in the intervention group, with no significant differences between control and intervention groups. The age group of 20–30 years showed highest proportion in both control and intervention groups at baseline and end line data.

Majority of the participants 659 (94%) at baseline and 638 (94.66%) at end line were Tegaru in ethnic group and 651 (93%) at baseline and 625 (92.73%) at end line were Orthodox followers and showed no significant differences between control and intervention groups at baseline (*p* value = 0.147 and 0.882) and end line (*p* value = 0.097 and 0.933) respectively. About 443 (63.3%) of the participants (59.7% in control and 66.8% in intervention) at baseline and 418 (62%) of the participants (58.68% in control and 65.3% in intervention) at end line were married. The variable marital status showed statistically significant differences both at baseline (*p* value = 0.003) and end line data (*p* value = 0.042).

Participants who had tertiary education were 87 (12.4%) at baseline (*p* value = 0.171) and 81 (12%) at end line (*p* value = 0.141). Housewife was highest proportion of the occupational status and showed no significant differences between control and intervention groups both at baseline (*p* value = 0.292) and end line data (*p* value = 0.175) (Table 1).

**Table 1:** Distribution of socio-demographic characteristics by treatment status at baseline and end line in Tigray, 2018

Socio-demographic characteristics	Baseline(***N*** = 700)				End line (***N*** = 674)			
	Control ***n*** (%)	Intervention ***n*** (%)	Total ***n*** (%)	***p***-value	Control ***n*** (%)	Intervention ***n*** (%)	Total ***n*** (%)	***p***-value
Age grouped								
20–30	177 (50.6)	170 (48.6)	347 (49.6)	0.926	174 (52.10)	151 (44.41)	325 (48.22)	0.221
31–40	110 (31.4)	113 (32.3)	223 (31.9)		105 (31.44)	118 (34.71)	223 (33.09)	
41–50	47 (13.4)	52 (14.9)	99 (14.1)		41 (12.28)	54 (15.88)	95 (14.09)	
51–60	16 (4.6)	15 (4.3)	31 (4.4)		14 (4.19)	17 (5.00)	31 (4.60)	
Mean age and ± SD	32.48 (± 8.9)	32.97 (± 8.5)		0.458	32.42 (± 9.03)		33.04 (± 8.44)	0.363
Ethnicity new1								
Tigray	334 (95.4)	325 (92.9)	659 (94.1)	0.147	321 (96.11)	317 (93.24)	638 (94.66)	0.097
Amhara	16 (4.6)	25 (7.1)	41 (5.9)		13 (3.89)	23 (6.76)	36 (5.34)	
Religion								
Orthodox	326 (93.1)	326 (93.1)	651 (93)	0.882	310 (92.81)	315 (92.65)	625 (92.73)	0.933
Muslim	24 (6.9)	24 (6.9)	49 (7)		24 (7.19)	25 (7.35)	49 (7.27)	
Marital status								
Married	209 (59.71)	234 (66.86)	443 (63.29)	0.003	196 (58.68)	222 (65.29)	418 (62.02)	0.042
Divorced or widowed	88 (25.14)	91 (26.00)	179 (25.57)		95 (28.44)	93 (27.35)	188 (27.89)	
Unmarried	53 (15.14)	25 (7.14)	78 (11.14)		43 (12.87)	25 (7.35)	68 (10.09)	
Educational status								
No formal education	92 (26.29)	72 (20.57)	164 (23.43)	0.171	90 (26.95)	71 (20.88)	161 (23.89)	0.141
Primary education	97 (27.71)	119 (34.00)	216 (30.86)		91 (27.25)	115 (33.82)	206 (30.56)	
Secondary education	115 (32.86)	118 (33.71)	233 (33.29)		110 (32.93)	116 (34.12)	226 (33.53)	
Tertiary education	46 (13.14)	41 (11.71)	87 (12.43)		43 (12.87)	38 (11.18)	81 (12.02)	
Occupation								
Civil servant	33 (9.43)	45 (12.86)	78 (11.14)	0.292	36 (10.78)	51 (15.00)	87 (12.91)	0.175
Merchant	68 (19.43)	77 (22.00)	145 (20.71)		67 (20.06)	78 (22.94)	145 (21.51)	
Housewife	128 (36.57)	129 (36.86)	257 (36.71)		124 (37.13)	125 (36.76)	249 (36.94)	
Daily worker	76 (21.71)	66 (18.86)	142 (20.29)		66 (19.76)	58 (17.06)	124 (18.40)	
Private employed	45 (12.86)	33 (9.43)	78 (11.14)		41 (12.28)	28 (8.24)	69 (10.24)	

### Reproductive health

Participants who had only one lifetime partner were 421 (60%) at baseline (60.3% in control and 60.1% in intervention) and 395 (58.6%) at end line data. About 370 (52.8%) of the participants at baseline and 369 (54.7%) at end line were contraceptive users. Both Lifetime partner and contraceptive users did not show statistical differences between control and intervention groups at baseline (*p* value = 0.208 and 0.289) and end line data (*p* value = 0.866 and 0.157) respectively.

Participants who had at least one abortion in their lifetime were 116 (16.57%) at baseline and 112 (16.6%) at end line. The variable did not show significant differences between control and intervention group at baseline (*p* value = 0.839) and end line (*p* value = 0.918) (Table 2).

**Table 2:** Distribution of reproductive health and risk factors of cervical cancer by treatment status of women over time in Tigray, 2018

Reproductive health variable	Baseline(***N*** = 700)				End line (***N*** = 674)			
	Control ***n*** (%)	Intervention ***n*** (%)	Total ***n*** (%)	***p***-value	Control ***n***(%)	Intervention ***n***(%)	Total ***n*** (%)	***p***-value
Life time partner								
Only one	210 (60.00)	211 (60.29)	421 (60.14)	0.208	199 (59.58)	196 (57.65)	395 (58.61)	0.866
> = 2 formal	99 (28.29)	111 (31.71)	210 (30.00)		101 (30.24)	109 (32.06)	210 (31.16)	
> = 2 informal	41 (11.71)	28 (8.00)	69 (9.86)		34 (10.18)	35 (10.29)	69 (10.24)	
Contraceptive use								
Yes	192 (54.86)	178 (50.86)	370 (52.86)	0.289	192 (57.49)	177 (52.06)	369 (54.75)	0.157
No	158 (45.14)	172 (49.14)	330 (47.14)		142 (42.51)	163 (47.94)	305 (45.25)	
Pregnancy								
1 to 4 pregnancy	266 (76.00)	270 (77.14)	536 (76.57)	0.070	248 (74.25)	254 (74.71)	502 (74.48)	0.133
5 or more pregnancy	55 (15.71)	65 (18.57)	120 (17.14)		61 (18.26)	72 (21.18)	133 (19.73)	
No pregnancy	29 (8.29)	15 (4.29)	44 (6.29)		25 (7.49)	14 (4.12)	39 (5.79)	
Abortion								
One or more abortion	59 (16.86)	57 (16.29)	116 (16.57)	0.839	56 (16.77)	56 (16.47)	112 (16.62)	0.918
No abortion	291 (83.14)	293 (83.71)	584 (83.43)		278 (83.23)	284 (83.53)	562 (83.38)	
**Risk factor variables**								
Age at first sex								
< = 20	302 (86.29)	314 (89.71)	616 (88.00)	0.163	289 (86.53)	306 (90.00)	595 (88.28)	0.161
> = 21	48 (13.71)	36 (10.29)	84 (12.00)		45 (13.47)	34 (10.00)	79 (11.72)	
Smoking								
Yes	31 (8.86)	19 (5.43)	50 (7.14)	0.078	36 (10.78)	23 (6.76)	59 (8.75)	0.065
No	319 (91.14)	331 (94.57)	650 (92.86)		298 (89.22)	317 (93.24)	615 (91.25)	
Alcohol use								
Drink always	27 (7.71)	19 (5.43)	46(6.57)	0.000	32 (9.58)	21 (6.18)	53 (7.86)	0.000
Drink sometimes	173 (49.43)	23 3 (66.57)	406 (58.00)		161 (48.20)	228 (67.06)	389 (57.72)	
No alcohol use	150 (42.86)	98 (28.00)	248 (35.43)		141 (42.22)	91 (26.76)	232 (34.42)	
STI history								
Yes	62 (17.71)	46 (13.14)	108 (15.43)	0.094	75 (22.46)	73 (21.47)	148 (21.47)	0.758
No	288 (82.29)	304 (86.86)	592 (84.57)		259 (77.54)	267 (78.53)	526 (78.04)	
Corticosteroid use								
Yes	30 (8.57)	21 (6.00)	51 (7.29)	0.191	30 (8.98)	30 (8.82)	60 (8.90)	0.942
No	320 (91.43)	329 (94.00)	649 (92.71)		304 (91.02)	310 (91.18)	614 (91.10)	

NB: *STI* sexually transmitted infection

### Risk factors of cervical cancer

In the majority of the participants, 616 (88%) at baseline (86.3% in control and 89.7% in intervention) and 595 (88.3%) at end line (86.5% in control and 90% in intervention) had first sexual contact before the age of 20 years. This variable did not show significant differences between control and intervention groups at baseline (*p* value = 0.163) and end line data (*p* value = 0.161).

About 50 (7.1%) participants at baseline and 59 (8.75%) at end line were smokers and had no significant differences between control and intervention groups at baseline (*p* value = 0.078) and end line (*p* value = 0.065). About 406 (58%) participants at baseline and 389 (57.7%) at end line used alcohol sometimes and showed significant differences between control and intervention groups at baseline (*p* value = 0.000) and end line (*p* value = 0.000).

Among participants, 108 (15.4%) at baseline and 148 (21.5%) at end line had history of sexually transmitted infection (STI) and showed no significant differences between control and intervention at baseline (*p* value = 0.094) and end line (*p* value = 0.758), (Table 2).

The impact of health education intervention on demand of women for cervical cancer screening. At baseline, all the outcome variables except “having plan to screen” showed no statistically significant differences between intervention and control groups. But at end line, all outcome variables showed statistically significant differences between intervention and control groups.

The proportion of participants who had willingness for cervical cancer screening was increased from 147 (42%) in the control and 135 (38.57%) in the intervention groups at baseline to 173 (51.8%) in control and 289 (85%) in the intervention group, at end line (*p* value = 0.000).

The proportion of participants who had a plan for screening was 44 (12.57%) in control and 72 (20.57%) in the intervention group at baseline (*p* value = 0.004), which increased to 63 (18.86%) in control and 141 (41.47%) in the intervention group at end line (*p* value = 0.000).

At baseline, 18 (5%) of the participants in control and 27 (7.7%) in the intervention group were screened for cervical cancer; with not statisticaly significant difference (*p* value = 0.165). At end line, participants who were screened were increased to 91 (27.3%) in control and 159 (46.8%) in the intervention group, with statistically significant difference (*p* value = 0.000).

**Fig. 4:**
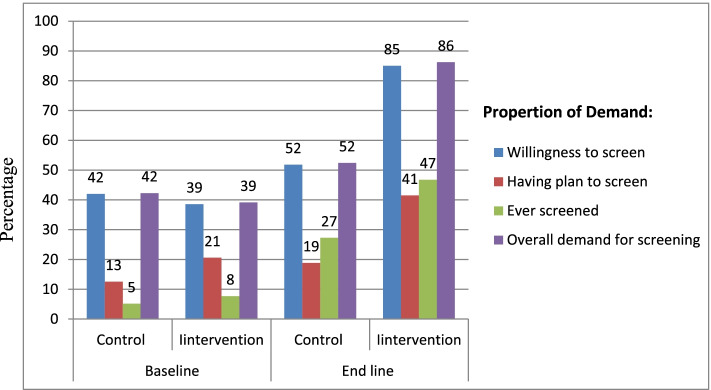
The proportion of demand of women for cervical cancer screening in intervention and control groups over time

At baseline, overall demand of participants for cervical cancer screening was 148 (42.3%) in control and 137 (39.14%) in the intervention group, with no statistical differences (*p* value = 0.397). However, the proportion of overall demand was increased to 175 (52.4%) in control and 293 (86.2%) in the intervention group at end line with statistically significant differences (both *p* value = 0.000) (Fig. 4).

### The difference in differences of demand for cervical cancer screening

The difference in lost to follow-up rates between control and intervention groups was 1.7%, hence, a per-protocol (PP) analysis is appropriate, though intent-to-treat analysis was also considered to see the effect of loss to follow-up on the difference in differences. Table 3 shows the difference in differences in proportion of demand between control and intervention groups over time.

**Table 3:** The estimation of difference in differences on demand of participants for cervical cancer screening over time, in Tigray, 2018

Outcome variables.		Baseline				End line				DID	
		Intervention %	Control %	% diff	***p***-value	Intervention %	Control %	% diff	***p***-value	%	***p***-value
Willingness	PP	38.6	42.0	− 3.4	0.328	85.0	51.8	33.2	0.000	36.6	0.000
	ITT	38.6	42.0	− 3.4	0.333	82.6	49.4	33.1	0.000	36.5	0.000
Plan to screen	PP	20.6	12.6	8.0	0.010	41.5	18.9	22.6	0.000	14.6	0.001
	ITT	20.6	12.6	8.0	0.009	40.3	18.0	22.3	0.000	14.3	0.001
Ever screened	PP	7.7	5.1	2.6	0.365	46.8	27.2	19.5	0.000	16.9	0.000
	ITT	7.7	5.1	2.6	0.364	45.4	26.0	19.4	0.000	16.8	0.000
Overall demand	PP	39.1	42.3	− 3.1	0.368	86.2	52.4	33.8	0.000	36.9	0.000
	ITT	39.1	42.3	− 3.1	0.373	83.7	50.0	33.7	0.000	36.9	0.000

The difference in differences in the proportion of participants who had willingness for cervical cancer screening between control and intervention groups over time was 36.6% (PP) (*p* value = 0.000) or 36.5 (ITT) (*p* value = 0.000). The difference in differences in proportion of participants who had plan for cervical cancer screening between control and intervention groups over time was 14.6% (PP) (*p* value = 0.001) or 14.3% (ITT), (*p* value = 0.001).

The difference in differences in proportion of participants who had been screened for cervical cancer between control and intervention group over time was 16.9% (PP) (*p* value = 0.000) or 16.8% (ITT), (*p* value = 0.000).

Finally, the difference in differences in the proportion of overall demand for cervical cancer screening between control and intervention groups over time was 36.9% (for both PP and ITT) (*p* value = 0.000).

**Fig. 5:**
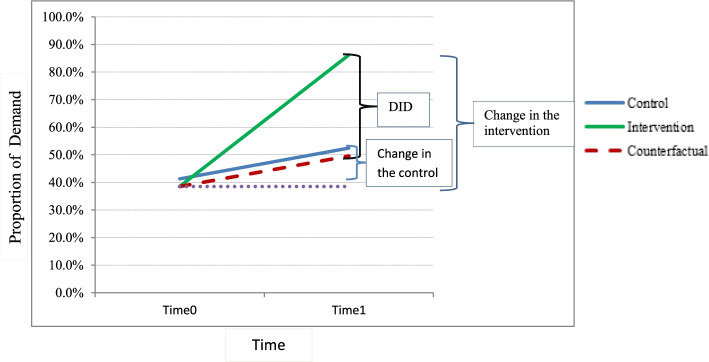
The difference in differences of the proportion of demand for cervical cancer screening among both groups in pre and post intervention

After follow-up, the intervention group showed more change in average predicted probability of demand for cervical cancer screening than the control group. The collapse command of the two by two table implies that the intervention line showed an increase in proportion of overall demand for cervical cancer screening greater than the increase in proportion of overall demand in the control group, (Fig. 5).

The random effect model complements that providing health education intervention increased the proportion in overall demand of women for cervical cancer screening by 36.9%, (95% C.I. (0 .29, 0.44), *p* value = 0.000) relative to those who did not get the health education intervention, Hence, the equation implies:

$${\mathrm{Demand}}_{est}=0.42-0.03\mathrm{Treated}+0.11\mathrm{Period}+0.37\mathrm{Treated}\ast \mathrm{Period}$$

## Discussion

This study was aimed to evaluate the impact of health education intervention on demand of adult women for cervical cancer screening in Tigray region. A structured power point presentation plus 6-month health extension workers consultation sessions were used as an intervention package. Outcome variables were measured at baseline and end line period and compared among intervention and control groups.

Using PP and ITT analysis, the proportion of demand did not show significant differences between control and intervention groups at baseline. But, after follow-up, this study showed significant increase in the proportion of demand for cervical cancer screening and the increase was higher in the intervention group than the control group, implying that health education intervention was effective. Providing health education enable participants to understand the benefits and availabilities of screening services. In many developing countries, reasons for a low cervical cancer screening service utilization were having poor knowledge on cervical cancer and its prevention method [7, 8, 11].

After follow-up of this study, willingness for cervical cancer screening was increased by 46.4% in the intervention group, which is greater than two studies done in Nigeria (1.5 and 1.7%) but less than a study done in Ghana (58.4%) [6, 7, 32]. The proportion of participants who had plan to screen within the next 6 months was also increased by 20.9% in the intervention group, which is greater than a study done in Ghana (13.4%) but less than a study done in North America (72%) [16, 32]. This might be the fact that the pre-intervention experience of participants could determine the outcome of intervention; hence, 84.2% of participants in the study from Ghana had knowledge on cervical cancer before intervention. In addition to the contextual differences, the intervention used in a study from America include tailored counseling and logistic assistance during home visit, which could motivate for the attendance of screening.

After follow-up, the screening practice in this study was increased to 46.8% in the intervention group greater than the control group (27.2%). Though this is less than studies done in Greece (88.1%) and Forsyth County (87%) [4, 15], the change in screening practice of the intervention group was 39.1%, which is greater than two studies done in Nigeria (6.8% and 4%), and Forsyth County (14%) [6, 7, 15]. The difference in differences of the proportion of screening practice in this study was 16.9%, which is greater than a studies done in Nigeria (3.6%) and America (10.6%), and almost similar with a study done in Forsyth County (17%), and less than a study done in Korea (22.2%) [7, 10, 15, 33]. The post intervention proportion of screening practice depends on the pre-intervention screening practice in the same group, the type of intervention, and duration of follow-up. An effective intervention implies more change in proportion of screening practice.

In this study, statistically significant difference in the overall demand was observed in the intervention groups than in the control group; with a difference in differences of 36.9%, which is greater than results from Barcelona (11%), Canada (12.3%), North America (24%), and Kenya (17.3%) [5, 12, 16, 34].

This difference might be due to the fact that our study was used a structured power point based face to face presentation followed by 6-month health extension workers consultation, which might provide an opportunity for inquiry and better understanding of the participants about cervical cancer screening than the other studies that were used sending invitation letter, phone call, informative leaflet, video show, pamphlet and brochure distribution, outreach campaign, and questionnaire administration as intervention methods. Besides, our study used the health extension workers who are working at the grass root level in the community, and this provides an opportunity for participants to have frequent contact in contrast to the other studies.

Though many studies showed an increase in cervical cancer screening practice following health education intervention, there were also studies conducted in Cameroon, Enugu state, Canada, and Portugal, which showed no statistically significant increases in the proportion of cervical cancer screening practice [8, 11, 12, 35]. This could be due to the fact that the intervention did not include in person communication; rather, it was like video show, self-administrated questionnaire, text messages, phone calls, and other multimodal community interventions that hinder the possibility of inquiry for unclear content and procedures. Structured health education, including a face to face presentation, followed by peer group discussion, and follow-up consultation with health extension workers for 6 month promote the inquiry for unclear content and procedures, which enabled them to make informed decisions about cervical cancer screening.

## Strength and limitation

This study was started immediately after launching the national cervical cancer prevention and control in 2015 in Ethiopia, which is timely and in line with governmental policy. Though randomization was at cluster level; an individual level analysis was used, which may decrease variance estimates and thereby increase the likelihood of significant finding. Since one participant was measured twice in both groups, the parallel trend assumption test is not possible.

The DID analysis may have limited external validity for the participants that have significant differences in their characteristics.

## Conclusion

Our study revealed that health education intervention on cervical cancer could improve the proportion of willingness, having plan to screen within the next 6 months, and screening practice for cervical cancer after 6 months post intervention. Based on the difference in differences, the health education intervention showed significant increase in the overall demand for cervical cancer screening.

Conducting community-based health education intervention is quite feasible and acceptable by the community that signify good opportunity to implement the national strategies on reproductive organ cancer prevention and control. Demand creation about cervical cancer screening using health education by health professionals is important to the success of a cervical cancer prevention and control strategies and transformation plan. Health education would also be better off by including the health promotion component of cervical cancer prevention and control strategies at regional level, which could ensure good knowledge and attitude of eligible women to improve cervical cancer screening behavior.

## Supplementary Information

**Additional file 1.**

## Data Availability

All data generated or analyzed during this study are included in this manuscript and its Supplementary information files uploaded as protocol.

## Abbreviations

CRCT: Cluster-randomized controlled trial
DID: Difference in difference
HEW: Health extension worker
HPV: Human papilloma virus
ICC: Intra cluster correlation
ITT: Intention to treat
LPM: Linear probability model
Pap: Papanicolaou
PP: Per protocol
STI: Sexually transmitted infections
TAU: Treatment as usual
UNICEF: United Nations International Children’s Emergency Fund
